# Influenza in travelers from Germany returning from abroad: a retrospective case–control study

**DOI:** 10.1186/s12879-024-10008-9

**Published:** 2024-10-05

**Authors:** Thomas Theo Brehm, Fabian Shijaku, Ralf Krumkamp, Johannes Jochum, Armin Hoffmann, Michael Ramharter, Benno Kreuels

**Affiliations:** 1 I. Department of Internal Medicine, University Medical Center Hamburg-Eppendorf, Martinistraße 52, Hamburg, 20246 Germany; 2 Department of Clinical Infectious Diseases, Leibniz Lung Center, Research Center Borstel, Parkallee 35, Borstel, 23845 Germany; 3 German Center for Infection Research (DZIF), Partner Site Hamburg-Lübeck-Borstel-Riems, Hamburg, Germany; 4 Department of Infectious Disease Epidemiology, Bernhard-Nocht-Institute for Tropical Medicine, Hamburg, Germany; 5 Department of Tropical Medicine, Bernhard Nocht Institute for Tropical Medicine, Hamburg, Germany; 6 Institute of Medical Microbiology, Virology and Hygiene, University Medical Center Hamburg-Eppendorf, Hamburg, Germany; 7 Present Address: Department of Implementation Research, Bernhard Nocht Institute for Tropical Medicine, Bernhard-Nocht-Straße 74, Hamburg, 20359 Germany

**Keywords:** Respiratory infection, Influenza vaccination, Seasonality, South-East Asia, Superinfection, Morbidity

## Abstract

**Background:**

Influenza is the most common vaccine-preventable infection among travelers, affecting approximately one percent of those travelling to subtropical and tropical destinations.

**Methods:**

We analysed demographic, travel-related and clinical information from travelers diagnosed with influenza at our travel clinic between January 2015 and March 2020 and influenza-negative controls.

**Results:**

We included 68 travelers diagnosed with influenza and 207 controls. In total, 22.1% of influenza patients (*n* = 15) were older than 60 years and/or had comorbidities for which annual influenza vaccination is recommended, but only one had received an influenza vaccine. Patients with respiratory and musculoskeletal symptoms who presented during the German influenza season had the highest risk proportion of positive tests (54%, *n* = 25/46). Overall, three (4.4%) influenza patients were hospitalised, two (2.9%) received antiviral treatment, and eight (11.8%) received antibiotic therapy.

**Conclusions:**

Influenza occurs throughout the year in international travelers and can cause significant morbidity. Travelers with febrile illness should be tested for influenza, especially if they have respiratory or musculoskeletal symptoms, present during the local influenza season, or have travelled to South-East Asia. Influenza vaccination coverage among international travelers needs to be improved among high-risk individuals.

**Supplementary Information:**

The online version contains supplementary material available at 10.1186/s12879-024-10008-9.

## Introduction

On average, influenza affects 5 to 15% of the world’s population, resulting in approximately 3 to 5 million cases of severe illness and about 290,000 to 650,000 deaths each year [1, 2]. Approximately one percent of travelers to subtropical and tropical destinations are known to be infected with influenza viruses, making it the most commonly reported vaccine-preventable infection among travelers prior to the coronavirus disease 2019 (COVID-19) pandemic [3, 4]. Travel-associated outbreaks have been reported particularly among Hajj pilgrims [5] and cruise ship passengers [6]. As the volume of international travel and the proportion of high-risk individuals among international travelers continues to increase, the impact of influenza on traveler morbidity is growing [7]. In addition, travelers may introduce existing and novel influenza viruses into their home country or other countries [8]. The aim of this single-centre retrospective analysis was to analyse demographic, travel-related and clinical information of international travelers diagnosed with seasonal influenza at our travel clinic. We also aimed to identify risk factors for testing positive for influenza when presenting with febrile illness after international travel by comparing these patients with a control group without influenza infection.

## Materials and methods

### Study population

We identified all febrile international travelers, defined as having a temperature > 38.0 °C, either self-reported or measured in the travel clinic, who were tested for influenza virus infection by PCR at the Travel Clinic of the University Medical Center Hamburg-Eppendorf at the Bernhard Nocht Institute for Tropical Medicine in Hamburg, Germany, between January 2015 and March 2020. According to the diagnostic algorithm at our center, all international travelers with febrile illness receive influenza testing by PCR. Only patients with complete data were included in the analysis. As the incubation period of influenza is one to four days, we included patients in the final analysis who reported a maximum of four days between return from travel and onset of symptoms. To identify risk factors for testing positive for influenza, we included a control group of patients who also presented at our travel clinic with febrile illness after international travel but had a negative PCR test. We aimed to match each influenza case with three controls within the same time period. We did not group the patients by calendar year (January to December) but instead chose time periods from July to June of the following year in order to capture the entire influenza season in the Northern Hemisphere. This approach allows us to encompass a full influenza season in Germany within a 12-month period. If we identified more than three controls for an influenza case in the respective time period, controls were randomly selected. The influenza group and controls were compared for sex, age, symptoms at presentation, duration of travel, and region of travel. We grouped travel destinations into the six World Health Organization (WHO) regions African Region, Region of the Americas, South-East Asian Region, European Region, Eastern Mediterranean Region, and Western Pacific Region. Demographic and clinical data were retrieved from the electronic and paper-based patient records and documented in a pseudonymised manner using a web-based, centralised, password-protected clinical database management system (REDCap) [9, 10].

### Statistical analyses

Age of patients in the influenza and control groups is expressed as median and interquartile range (IQR) and compared by Mann–Whitney U test. Categorical variables are expressed as frequencies and percentages and compared by Fisher’s exact test. Conditional binary logistic regression was calculated to show associations between influenza diagnosis and categorical variables stratified by year of recruitment. An interaction term was included in the regression to show the effect of a risk factor within categories of a second variable. The regression coefficients of the interaction terms were linearly combined to present odds ratios (ORs) representing these combined effects. Patients whose region of travel was unknown were not included in the statistical analyses. Due to small sample sizes, the travel destinations Eastern Mediterranean region and European region were combined into a single category. A multivariate logistic regression model was used to identify independent predictive factors for testing positive for influenza. An a priori decision was made to include the variables age ≥ 60 years, male sex and age-adjusted Charlson Comorbidity Index (ACCI) in the model. Finally, a classification and regression tree (CART) was constructed based on the results of the multivariable regression analysis to translate the associations into a decision flowchart. Variables with effect estimates of OR < 0.5 or > 2.0 were included in the model. All data analyses were performed using R (version 4.3.1) with the packages survival (version 3.5–5) for calculating conditional logistic regression, multcomp (version 1.4–25) for combining regression coefficients, and rpart (version 4.1.19) for constructing the CART. Graphs were generated using GraphPad Prism, version 9 for MacOS (GraphPad Software, La Jolla, California, USA).

## Results

### Study population

**Fig. 1:**
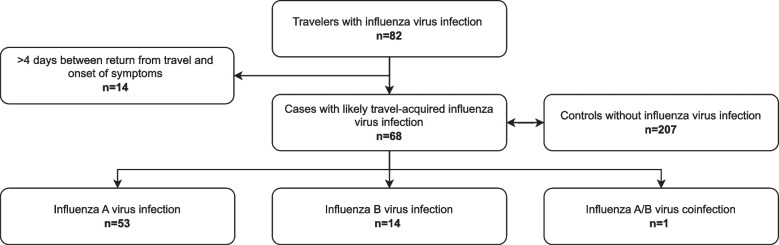
Selection of the study population and the control group. Legend: We included international travelers diagnosed with influenza at our travel clinic between January 2015 and March 2020. Those reporting more than four days between their return from travel and the onset of symptoms were excluded. To identify risk factors for testing positive for influenza among travelers presenting with febrile illness, we aimed to match influenza negative control patients who presented during the same time period at a 3:1 ratio

**Fig. 2:**
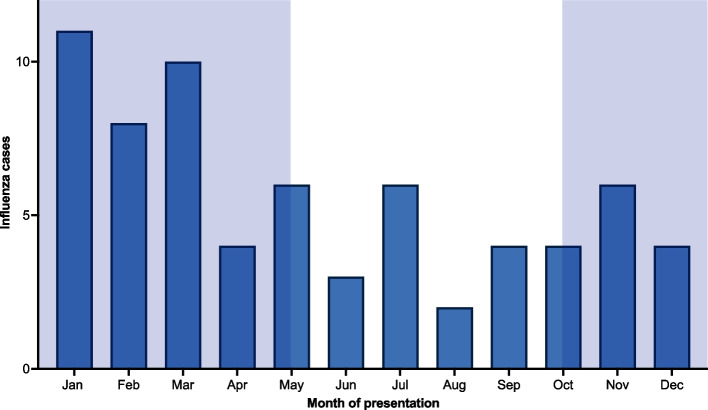
Monthly distribution of influenza cases. Legend: The number of patients diagnosed with influenza is shown per calendar month of presentation at our travel clinic. The German influenza season (calendar week 40 in early October to calendar week 20 in late May) is highlighted in light blue

During the study period, 82 international travelers were diagnosed with seasonal influenza virus infection. Fourteen of these were excluded from the final analysis because they reported more than four days between return from travel and onset of symptoms. Finally, 68 patients with seasonal influenza were included in the analysis (Fig. 1). Duration between onset of symptoms and return from travel was less than one day (*n* = 17; 25.0%), one day (*n* = 19; 27.9%), two days (*n* = 2; 2.9%), three days (*n* = 5; 7.4%), or four days (*n* = 4; 5.9%). Twenty patients (29.4%) had developed symptoms before returning to Germany. During the same time period, 438 patients were tested negative for influenza virus infection at our travel clinic after international travel. To identify potential risk factors for testing positive for influenza after international travel we selected a control group of 207 febrile travelers who tested negative for influenza as described above (Supplement S1). Overall, 27.9% (*n* = 19) of the influenza patients presented outside the influenza season in Germany, which runs from calendar week 40 in early October to calendar week 20 in late May. All these patients returned from countries with perennial influenza virus circulation. The detailed monthly distribution of influenza cases is shown in Fig. 2. In the control group, 42.5% (*n* = 88) presented outside the German influenza season. An interaction analysis was calculated for German influenza season and travel to South-East Asia, using the risk of positive influenza test outside the German season for patients not travelling to South-East Asia served as the reference group. Patients presenting during the German influenza season and travelling to South-East Asia showed the highest risk for a positive test result (OR = 4.27; 95% CI 1.62–11.28; *p* = 0.36), followed by travelers during the German influenza season with a destination outside South-East Asia (OR = 3.31; 95% CI 1.36–8.06; *p* = 0.01). An increased risk was also seen in those who travelled outside the German season, but to a destination within South-East Asia (OR = 2.44; 95% CI 0.89–6.73; *p* = 0.08).

### Demographic and travel-related characteristics

**Fig. 3:**
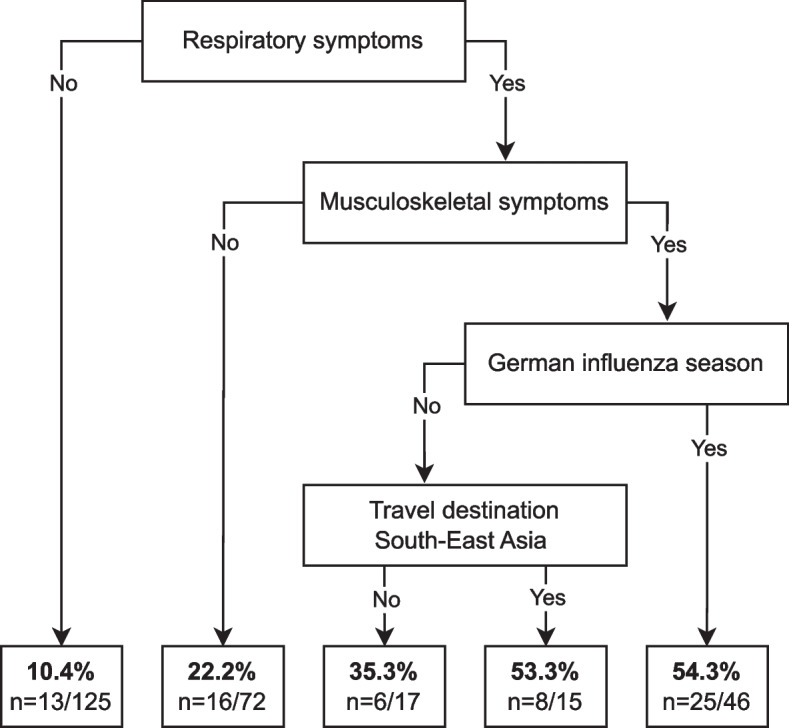
Decision flowchart generated by classification and regression tree (CART) analysis. Legend: Classification and regression tree (CART) was constructed based on the results of the multivariable regression analysis. Variables with effect estimates of OR < 0.5 or > 2.0 were included in the model

The median age in the influenza group was 33 years (IQR: 27–44 years), and 38.2% (*n* = 26) of the patients were female (Table 1). The majority of patients (77.9%; *n* = 53) were diagnosed with influenza A virus infections, which were subtyped as H1N1 (38.2%; *n* = 26) or H3N2 (35.3%; *n* = 24). Three patients with influenza A virus infections could not be subtyped. Fourteen patients (20.6%) were diagnosed with influenza B virus infections. One patient had a coinfection with influenza A virus (H3N2) and influenza B virus. The most common travel regions for influenza cases were the South-East Asian Region (42.6%; *n* = 29), the African Region (27.9%; *n* = 19), and the Region of the Americas (17.6%; *n* = 12). The majority (61.8%; *n* = 42) of travelers were tourists. Less common reasons for travelling were work (16.2%; *n* = 11) or visiting friends and relatives (VFR) (7.4%; *n* = 5). The duration of travel was most frequently up to one week (20.6%; *n* = 14), between one and two weeks (30.9%; *n* = 21) or two and three weeks (33.8%; *n* = 23). Travel modalities and travel duration were not associated with testing positive for influenza. The most common symptoms among cases were respiratory symptoms (76.5%; *n* = 52), musculoskeletal symptoms (69.1%; *n* = 47) and headache (55.9%; *n* = 38). Patients presenting with respiratory symptoms (OR 5.42; 95% CI 2.73–10.76; *p* < 0.001) and musculoskeletal symptoms (OR 2.33; 95% CI 1.29–4.20; *p* = 0.01) were more likely to test positive for influenza. Table 1 shows the results of the bivariable and multivariable regression models described above. The results of the multivariable regression did not change substantially from the bivariable estimates. The multivariable regression was used for the CART analysis, which is shown in Fig. 3. Travelers without respiratory symptoms (10.4%; *n* = 13/125) and those with respiratory but no additional musculoskeletal symptoms (22.2%; *n* = 16/72) had the lowest proportion of positive influenza test results. The highest proportion of positive tests was seen in patients with respiratory and musculoskeletal symptoms who travelled during the German influenza season (54.3%, *n* = 25/46) or who travelled outside the season but to a country in South-East Asia (53.3%, *n* = 8/15).

**Table 1:** Demographic, travel-related and clinical characteristics of international travelers with influenza and the control group and bivariate and multivariate analyses

			**Bivariate analysis**			**Multivariate analysis**		
	**Influenza group**	**Control group**	**OR**	**95% CI**	**p**	**OR**	**95% CI**	**p**
**n**	68	207						
Female sex	26 (38.2)	107 (51.7)	0.59	0.34–1.04	0.07	0.45	0.23–0.88	0.02
Male sex	42 (61.8)	100 (48.3)	Ref			Ref		
≥ 34 years of age	32 (47.1)	109 (52.7)	0.80	0.47–1.39	0.44	0.59	0.30–1.17	0.13
< 34 years of age	36 (52.9)	98 (47.3)	Ref			Ref		
Seasonality * SEA travel region interaction								
German influenza season, SEA region	49 (72.1)	119 (57.5)	4.27	1.62–11.28	0.36*	6.05	1.99–18.41	0.10*
German influenza season, outside SEA	19 (27.9)	88 (42.5)	3.31	1.36–8.06	0.01	5.52	2.01–15.18	< 0.001
No German influenza season, SEA region	29 (42.6)	69 (33.3)	2.44	0.89–6.73	0.08	3.04	0.93–9.89	0.06
No German influenza season, outside SEA	39 (57.4)	138 (66.7)	Ref			Ref		
Tourism	42 (61.8)	136 (65.7)	0.85	0.48–1.50	0.58	0.90	0.44–1.86	0.78
Other travel reason	26 (38.2)	71 (34.3)	Ref			Ref		
≥ 8 days travel duraction	54 (79.4)	181 (87.4)	0.61	0.29–1.27	0.11	0.43	0.17–1.10	0.08
< 8 days travel duration	14 (20.6)	26 (12.6)	Ref			Ref		
Respiratory symptoms	55 (80.9)	95 (45.9)	5.42	2.73–10.76	< 0.001	7.40	3.46–15.83	< 0.001
No respiratory symptoms	13 (19.1)	112 (54.1)	Ref			Ref		
Musculoskeletal symptoms	47 (69.1)	101 (48.8)	2.33	1.29–4.20	0.01	2.99	1.53–5.84	< 0.01
No musculoskeletal symptoms	21 (30.9)	106 (51.2)	Ref			Ref		

*Abbreviations: CI *Confidence interval,* SEA *South East Asia

*p–value of the interaction term

### Vaccination status

Among the influenza cases, only one patient had received an influenza vaccination before departure or during the last influenza season in Germany. A further 35 (51.5%) patients reported that they had not been vaccinated, and no information on influenza vaccination status was available for the remaining 32 (47.1%) patients. However, 22.1% (*n* = 15) of the travelers diagnosed with influenza were older than 60 years (2.9%; *n* = 2) and/or had comorbidities such as chronic pulmonary disease (8.8%; *n* = 6) or autoimmune diseases (7.4%; *n* = 5), for which annual vaccination against seasonal influenza is recommended by the Standing Committee on Vaccination (STIKO) of the Robert Koch Institute (RKI) in Germany (Supplement S2) [11]. In the control group, 4.3% (*n* = 9) of patients were vaccinated against influenza, 62.8% (*n* = 130) were unvaccinated, and for 32.9% (*n* = 68) of patients, no information on the influenza vaccination status was available.

### Clinical characteristics

Overall, 2.9% (*n* = 2) of the influenza patients received antiviral therapy with oseltamivir, and 11.8% (*n* = 8) received antibiotic treatment for suspected bacterial superinfection. Three male patients were hospitalised for one, six, and seven days, respectively, due to poor general clinical condition. They were 30, 50, and 78 years old and none of them required oxygen supplementation. None of the patients were admitted to the intensive care unit, and there were no deaths in our study cohort. The most common alternative diagnoses in the control group were bacterial gastroenteritis (*n* = 36; 17.4%), dengue fever (*n* = 14; 6.8%), upper respiratory tract infection (*n* = 9; 4.3%), urinary tract infection (*n* = 5; 2.4%), community-acquired pneumonia (*n* = 4; 1.9%), and Epstein–Barr virus (EBV) infection (*n* = 4; 1.9%) (Supplement S3).

## Discussion

In this retrospective case–control study, we report the demographic and clinical characteristics of 68 patients with travel-acquired influenza. Three patients were hospitalised, demonstrating the significant morbidity associated with influenza in international travelers. An important finding of our study is the low proportion of patients who were vaccinated against influenza before departure or during the previous influenza season. In Germany, the Standing Committee on Vaccination (STIKO) of the Robert Koch Institute (RKI) recommends seasonal influenza vaccination for all adults over 60 years of age, patients with certain chronic comorbidities, and pregnant women [11]. More than a fifth of cases in our study cohort met at least one of these criteria, but only a minority were vaccinated. This is consistent with the overall low uptake of seasonal influenza vaccination in the general population in Germany. In the 2021/22 season, only 43.3% of adults aged over 60 years, 35.4% of those with relevant comorbidities and 17.5% of pregnant women were vaccinated against seasonal influenza [12]. In addition to the above recommendations, many national and international societies advise all international travelers to be vaccinated against seasonal influenza to prevent both direct morbidity and onward transmission [13, 14]. We have previously conducted a questionnaire-based survey of travel health advisers in Germany regarding their attitudes, practices, and barriers to influenza vaccination of international travelers [15]. Although respondents reported that they generally recommend seasonal influenza vaccination to a significant proportion of international travelers throughout the year, very few respondents reported having regular access to influenza vaccines in June (6.5%), July (4.0%) and August (6.9%). Only 4.4% of respondents said they had ever ordered doses of influenza vaccine produced for the southern hemisphere to vaccinate travelers during the summer months in Germany. Among the influenza cases in our study cohort, only a minority had received pre-travel medical advice and only the single patient mentioned above had received influenza vaccination. In the current analysis, we cannot determine how often influenza vaccination was recommended by the travel health advisors but declined by the travelers and how often vaccination was not administered due to limited availability. Patients with febrile illness who also reported respiratory symptoms or musculoskeletal symptoms were significantly more likely to test positive for influenza than control patients. This finding is consistent with previously reported symptoms of seasonal influenza [16, 17]. However, it is important to note that malaria must always be excluded in all travelers returning from endemic regions with febrile illness. First, co-infection with malaria and influenza may occur [18, 19]. Second, malaria itself can cause influenza-like illness [20]. Interestingly, patients presenting during the German influenza season were more likely to test positive for influenza than patients presenting outside the German influenza season. This may reflect the risk of acquiring influenza infection not only at the travel destination, but also through close contact with other people in confined spaces after returning home, e.g. on the plane, at the airport or during land transport. In total, 27.9% (*n* = 19) of the patients who presented outside the influenza season in Germany returned from countries with perennial influenza virus circulation, so that infection at the travel destination is likely. Furthermore, the CART analysis showed a comparable proportion of positive test results in patients with influenza symptoms who were traveling during the German influenza season and in patients with influenza symptoms who returned from South-East Asia but traveling outside the German influenza season.

Our study has several limitations. First, we only included patients who presented to our travel clinic with symptomatic febrile illness and were subsequently diagnosed with influenza. Therefore, unlike prospective seroprevalence studies [3, 16], we are unable to determine incidence rates or identify risk factors for influenza virus infection during international travel. Patients with asymptomatic infection, those with milder illness who may not have sought medical attention, and those who became symptomatic during travel and have sought medical attention at the travel destination would have been missed. However, our study is valuable in characterising the demographic, travel-related and clinical information of travelers presenting with influenza and in identifying risk factors for testing positive for influenza in patients presenting with febrile illness after international travel. Second, we do not know with absolute certainty that all patients in our cohort were infected during international travel. Although we only included patients who reported a maximum of four days between return from travel and onset of symptoms with regard to the incubation period of influenza, some patients may have become infected after their return to Germany, especially during the local influenza season. While 27.9% (*n* = 19) of the influenza patients presented outside the influenza season in Germany, the remaining patients theoretically may also have acquired autochthonous infections. A total of 275 patients were included in the study; however, this sample size may not be sufficient to provide robust estimates for subgroup analyses. This limitation should be considered when interpreting the effects of combined risk factors, as calculated in the CART analysis, where group results are based on small case numbers.

## Conclusion

Infections with seasonal influenza viruses occur throughout the year in international travelers and can cause significant morbidity. Travelers with febrile illness should be tested for influenza, especially those with respiratory or musculoskeletal symptoms, who travel during the local influenza season or who have visited South-East Asia. There is a pressing need to increase influenza vaccination coverage among international travelers, focusing on those at higher risk of severe disease.

## Supplementary information

Supplementary Material 1.

Supplementary Material 2.

Supplementary Material 3.

## Data Availability

The data that support the findings of this study are available from the corresponding author upon reasonable request.

## Abbreviations

ACCI: Age-adjusted Charlson Comorbidity Index
CART: Classification and regression tree
IQR: Interquartile range
OR: Odds ratio
