# Supplementary material for: Influenza in travelers from Germany returning from abroad: a retrospective case–control study
Source: BMC Infect Dis. 2024 Oct 5;24:1107. doi: 10.1186/s12879-024-10008-9 (PMC11453041; doi:10.1186/s12879-024-10008-9)
Supplement: Supplementary file 1 — Supplementary Material 1. [file 12879_2024_10008_MOESM1_ESM.docx]

**S3. Monthly distribution of the influenza group and the control group**

To identify risk factors for testing positive for influenza, a control group of patients who presented with febrile illness after international travel but tested negative by PCR was included. Each influenza case was matched with three controls within the same time period. Instead of using calendar years, we grouped patients by influenza season (July to June of the following year) to ensure we captured the full influenza season in Germany.
