# Supplementary material for: Influenza in travelers from Germany returning from abroad: a retrospective case–control study
Source: BMC Infect Dis. 2024 Oct 5;24:1107. doi: 10.1186/s12879-024-10008-9 (PMC11453041; doi:10.1186/s12879-024-10008-9)
Supplement: Supplementary file 2 — Supplementary Material 2. [file 12879_2024_10008_MOESM2_ESM.docx]

**Supplemental Table 1.** Comorbidities of patients in the influenza group and the control group

| **Comorbidities, n (%)** | **Influenza group** | **Control group** |
| --- | --- | --- |
| Total | 15 (22.1) | 20 (9.7) |
| Chronic pulmonary disease | 6 (8.8) | 6 (2.9) |
| Autoimmune disease | 5 (7.4) | 4 (1.9) |
| Hematooncological disease | 5 (7.4) | 4 (1.9) |
| Cardiovascular disease | 3 (4.4) | 4 (1.9) |
| Chronic kidney disease | 1 (1.5) | 0 |
| Immunosuppression | 1 (1.5) | 0 |
| Chronic liver disease | 0 | 1 (0.5) |
