# Supplementary material for: Influenza in travelers from Germany returning from abroad: a retrospective case–control study
Source: BMC Infect Dis. 2024 Oct 5;24:1107. doi: 10.1186/s12879-024-10008-9 (PMC11453041; doi:10.1186/s12879-024-10008-9)
Supplement: Supplementary file 3 — Supplementary Material 3. [file 12879_2024_10008_MOESM3_ESM.docx]

| **Alternative diagnosis, n (%)** |  |
| --- | --- |
| Bacterial gastroenteritis | 36 (17.4) |
| Dengue fever | 14 (6.8) |
| Upper respiratory tract infection | 9 (4.3) |
| Urinary tract infection | 5 (2.4) |
| Community-acquired pneumonia | 4 (1.9) |
| Epstein–Barr virus (EBV) infection | 4 (1.9) |
| Typhoid fever | 3 (1.4) |
| Malaria | 2 (1.0) |
| Cytomegalovirus (CMV) infection | 2 (1.0) |
| Pyelonephritis | 2 (1.0) |
| Soft tissue infection | 2 (1.0) |
| Tonsillitis | 2 (1.0) |
| Rickettsiosis | 1 (0.5) |
| Scrub typhus | 1 (0.5) |
| Sinusitis | 1 (0.5) |
| Lyme disease | 1 (0.5) |
| Chikungunya | 1 (0.5) |
| Giardiasis | 1 (0.5) |
| No diagnosis | 116 (56.0) |

**Supplemental Table 2.** Alternative diagnoses among control patients
